# Hématome mammaire: complication du traitement antithrombotique

**DOI:** 10.11604/pamj.2015.20.325.6376

**Published:** 2015-04-06

**Authors:** Houssine Boufettal, Naïma Samouh

**Affiliations:** 1 Service de Gynécologie-Obstétrique « C », Centre Hospitalier Universitaire Ibn Rochd, Faculté de Médecine et de Pharmacie, Université Hassan II, Casablanca, Maroc

**Keywords:** Hématome mammaire, antithrombotique, prothèse, ecchymoses, Breast hematoma, antithrombotic therapy, prosthesis, ecchymosis

## Image en medicine

Une patiente âgée de 57 ans, était suivie depuis 14 ans pour un rétrécissement mitral serré avec remplacement valvulaire mitral par prothèse mécanique. Elle était sous anticoagulants, type Acénocoumarol (Sintroms^®^) à la dose de 6 mg/jour avec un INR dans la zone thérapeutique. Elle présentait brutalement une tuméfaction brutale du sein gauche avec ecchymoses étendues aux deux seins et du dos. A son admission, la patiente était consciente avec un score de Glasgow du coma à 15. La tension artérielle était à 95/65 mmHg, la fréquence respiratoire à 14 cycles/min et les conjonctives légèrement décolorées. L'examen général ne retrouvait pas de saignement extériorisé. L'examen abdominal était normal. L'hémogramme retrouvait une hémoglobine à 5,4 g/dl et des plaquettes normales. Le taux de prothrombine était à 12% et l'INR à 7,8. Une échographie mammaire objectivait un hématome de tout le sein gauche et refoulant le tissu glandulaire en arrière. La correction des troubles de l'hémostase consistait en l'arrêt du traitement antivitamine K et l'injection de 2 mg de vitamine K en intraveineux direct. Un ajustement du bilan d'hémostase était obtenu. L'hématome mammaire était respecté. Une surveillance de la patiente montrait une résorption progressive de l'hématome au bout de trois mois. Aucune complication n’était survenue. Avec un recul de 24 mois, aucune récidive n’était notée.

**Figure 1:**
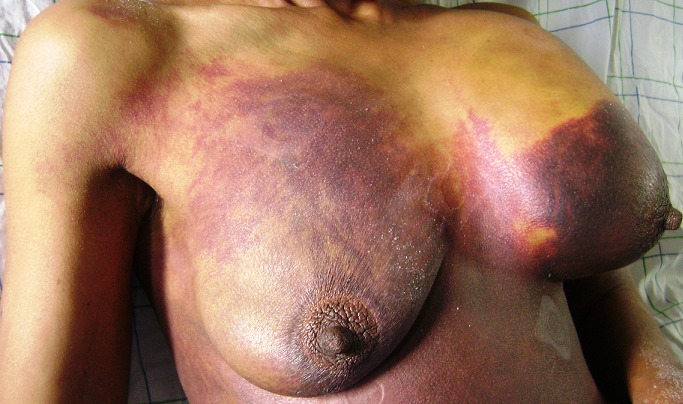
Tuméfaction du sein gauche secondaire à un hématome intramammaire, avec ecchymoses étendus aux deux seins et tronc, chez une patiente sous anti vitamine K

